# New Insights into Hepatic and Intestinal Microcirculation and Pulmonary Inflammation in a Model of Septic Shock and Venovenous Extracorporeal Membrane Oxygenation in the Rat

**DOI:** 10.3390/ijms25126621

**Published:** 2024-06-16

**Authors:** Fabian Edinger, Thomas Zajonz, Lena Holtz, Götz Schmidt, Emmanuel Schneck, Michael Sander, Christian Koch

**Affiliations:** Department of Anesthesiology, Operative Intensive Care Medicine and Pain Therapy, University Hospital, Justus-Liebig-University, 35392 Giessen, Germany

**Keywords:** V-V ECMO, perfusion, sepsis

## Abstract

Treatment of critically ill patients with venovenous (V-V) extracorporeal membrane oxygenation (ECMO) has gained wide acceptance in the last few decades. However, the use of V-V ECMO in septic shock remains controversial. The effect of ECMO-induced inflammation on the microcirculation of the intestine, liver, and critically damaged lungs is unknown. Therefore, the aim of this study was to measure the hepatic and intestinal microcirculation and pulmonary inflammatory response in a model of V-V ECMO and septic shock in the rat. Twenty male Lewis rats were randomly assigned to receive V-V ECMO therapy or a sham procedure. Hemodynamic data were measured by a pressure-volume catheter in the left ventricle and a catheter in the lateral tail artery. Septic shock was induced by the intravenous infusion of lipopolysaccharide (1 mg/kg). During V-V ECMO therapy, rats received lung-protective ventilation. The hepatic and intestinal microcirculation was assessed by micro-lightguide spectrophotometry after median laparotomy for 2 h. Systemic and pulmonary inflammation was measured by enzyme-linked immunosorbent assays of plasma and bronchoalveolar lavage (BAL), respectively, which included tumor necrosis factor alpha (TNF-α), interleukin 6 (IL-6), IL-10, C-X-C motif ligand 2 (CXCL2), and CXCL5. Reduced oxygen saturation and relative hemoglobin concentration were measured in the hepatic and intestinal microcirculation during treatment with V-V ECMO. These animals also showed increased systolic, mean, and diastolic blood pressures. While no differences in left ventricular ejection fraction were observed, animals in the V-V ECMO group presented an increased heart rate, stroke volume, and cardiac output. Blood gas analysis showed dilutional anemia during V-V ECMO, whereas plasma analysis revealed a decreased concentration of IL-10 during V-V ECMO therapy, and BAL measurements showed increased concentrations of TNF-α, CXCL2, and CXCL5. Rats treated with V-V ECMO showed impaired microcirculation of the intestine and liver during septic shock despite increased blood pressure and cardiac output. Despite lung-protective ventilation, increased pulmonary inflammation was recognized during V-V ECMO therapy in septic shock.

## 1. Introduction

The first prolonged treatment of a patient suffering from acute respiratory distress syndrome (ARDS) with venovenous extracorporeal membrane oxygenation (V-V ECMO) was described in 1972 [1]. Although V-V ECMO therapy was only used in very few and well-selected patients for several years, its global use has increased over the last two decades for several reasons. The CESAR trial provided evidence for the positive effects of V-V ECMO therapy on severe ARDS patients, and the 2009 influenza pandemic and severe acute respiratory syndrome coronavirus 2 pandemic in 2020 were associated with the increasing application of V-V ECMO as a rescue therapy [2,3].

The current guidelines of the Surviving Sepsis Campaign define sepsis as a dysregulated host response to infection resulting in life-threatening organ dysfunction [4]. Although many efforts have been made to improve the management of sepsis and septic shock, mortality remains high [5].

However, the use of V-V ECMO therapy in sepsis remains controversial. In this context, the large foreign surface area of the circuit and membrane leads to endothelial damage and the activation of the complement, inflammatory, and coagulation systems, a phenomenon known as ECMO-induced inflammation [6].

During V-V ECMO therapy, highly oxygenated blood is pumped through critically damaged lungs. The supraphysiological oxygen concentrations can cause the production of reactive oxygen species, which are known to induce pulmonary cell death [7]. Hyperoxemia in severe infection is shown to be associated with increased mortality [8]. In this context, the impact of the highly oxygenated blood on pulmonary inflammation during septic shock is unclear.

During sepsis, pathogen-associated molecular patterns lead to systemic inflammation and septic shock [9]. Loss of the tight junctions between endothelial cells is associated with increased capillary permeability and decreased perfusion of the tissues [10]. In the intestine, this phenomenon can lead to the translocation of intestinal bacteria into the blood. As a matter of fact, the intestine is also known as the “motor of sepsis” [11]. However, the impact of ECMO-induced inflammation on the intestinal barrier during septic shock is unknown.

Therefore, the primary aim of this study was to investigate the intestinal microcirculation during V-V ECMO therapy in a rat model of septic shock. Its secondary aim was to evaluate the inflammatory response of the lung during sepsis in rats undergoing ECMO therapy.

## 2. Results

### 2.1. Intestinal and Hepatic Microcirculation

**Figure 1:**
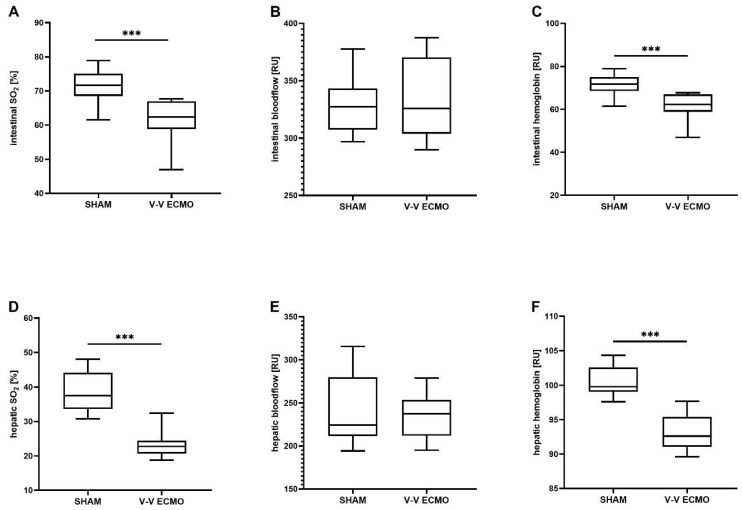
Means of the time course of (**A**) regional oxygen saturation, (**B**) relative blood flow, and (**C**) relative hemoglobin of the intestine and (**D**) regional oxygen saturation, (**E**) relative blood flow, and (**F**) relative hemoglobin of the liver. Animals treated with V-V ECMO showed reduced regional oxygen saturation and relative hemoglobin of the intestine and liver compared to sham animals. The asterisks denote the degree of statistical significance (*** *p* < 0.001). Abbreviations: ECMO = extracorporeal membrane oxygenation; RU = relative units; SO_2_ = regional oxygen saturation; V-V = venovenous.

White light and laser Doppler spectrometry of the intestine revealed a reduced SO_2_ (*p* < 0.001) and relative hemoglobin concentration (*p* < 0.001) in animals treated with V-V ECMO (Figure 1A,C). Furthermore, measurements of the liver showed a reduced regional oxygen saturation (SO_2_; *p* < 0.001) and relative hemoglobin level (*p* < 0.001) during V-V ECMO therapy (Figure 1D,F). However, measurements of intestinal and hepatic relative blood flow revealed no differences between sham- and V-V ECMO-treated animals (Figure 1B,E).

### 2.2. Hemodynamic Parameters

**Figure 2:**
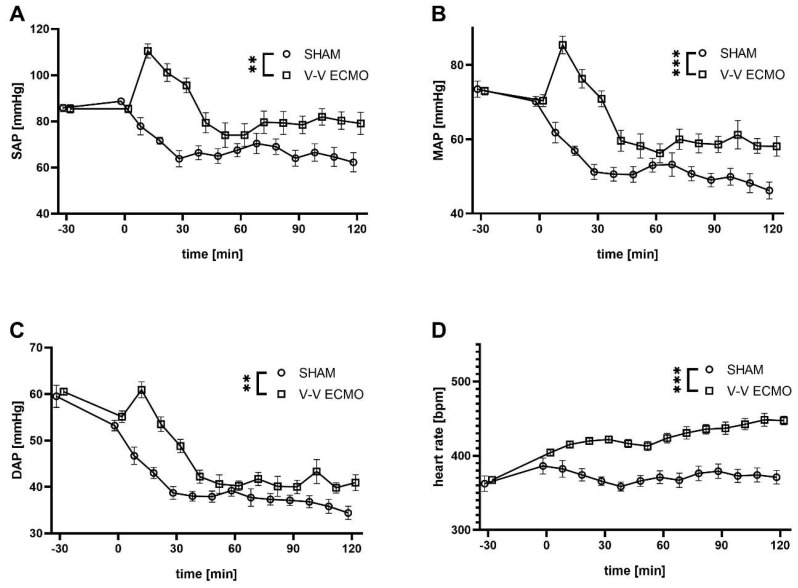
Time course of (**A**) systolic, (**B**) mean and (**C**) diastolic blood pressure and (**D**) heart rate. Animals treated with V-V ECMO showed elevated systolic, mean, and diastolic blood pressures compared to sham animals. Further, an increased heart rate was measured during V-V ECMO therapy. The asterisks denote the degree of statistical significance (** *p* < 0.01; *** *p* < 0.001). Abbreviations: DAP = diastolic arterial pressure; ECMO = extracorporeal membrane oxygenation; MAP = mean arterial pressure; SAP = systolic arterial pressure; V-V = venovenous.

Animals treated with V-V ECMO showed increased systolic, mean, and diastolic arterial blood pressures (*p* = 0.002, *p* < 0.001, and *p* = 0.001, respectively) compared to the sham group (Figure 2). Further, an increased cardiac rate was measured during V-V ECMO therapy (*p* < 0.001; Figure 2).

**Figure 3:**
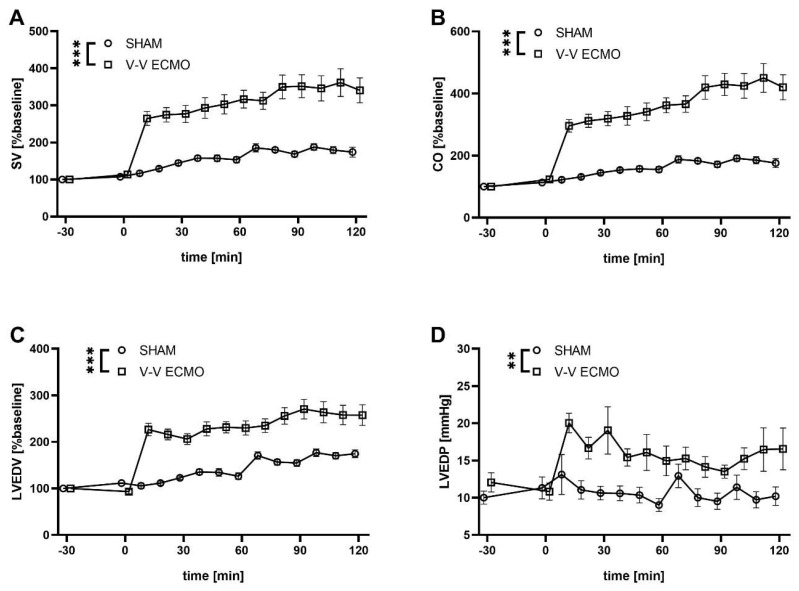
Time course of (**A**) stroke volume (SV), (**B**) cardiac output (CO), (**C**) left ventricular end-diastolic volume (LVEDV), and (**D**) left ventricular end-diastolic pressure (LVEDP). Animals treated with V-V ECMO showed increased SV, CO, LVEDV and LVEDP compared to sham animals. The asterisks denote the degree of statistical significance (** *p* < 0.01; *** *p* < 0.001). Abbreviations: CO = cardiac output; ECMO = extracorporeal membrane oxygenation; LVEDP = left ventricular end-diastolic pressure; LVEDV = left ventricular end-diastolic volume; SV = stroke volume; V-V = venovenous.

Moreover, analysis of the pressure-volume data demonstrated an increased stroke volume (SV; *p* < 0.001), cardiac output (CO; *p* < 0.001), left ventricular end-diastolic volume (LVEDV; *p* < 0.001), and pressure (LVEDP; *p* = 0.002) in animals treated with V-V ECMO compared to the sham group (Figure 3).

Although the time course of left ventricular ejection fraction (LVEF) showed a trend toward reduction in the sham compared to the V-V ECMO group after the end of the experiments, the difference failed to reach statistical significance (*p* = 0.053).

### 2.3. Blood Gas Analysis

No differences were found over time between animals treated with V-V ECMO and the sham group regarding central venous oxygen saturation (S_cv_O_2_; *p* = 0.321), arterial oxygen partial pressure (pO_2_; *p* = 0.360), lactate (*p* = 0.427), and sodium (*p* = 0.216; Table 1). Animals in the sham group presented a lower arterial oxygen saturation (S_a_O_2_; *p* = 0.042), hematocrit (*p* < 0.001), hemoglobin (*p* < 0.001), and chloride concentration (*p* = 0.013). Furthermore, elevated levels of arterial carbon dioxide partial pressure (pCO_2_; *p* < 0.001) and, consecutively, reduced pH (*p* < 0.001), bicarbonate (*p* < 0.001), and base excess (*p* < 0.001) were measured in these animals (Table 1). Moreover, increased concentrations of glucose (*p* = 0.033), potassium (*p* = 0.001) and calcium (*p* = 0.035) were measured in animals in the sham group (Table 1).

### 2.4. Inflammatory Parameters

**Figure 4:**
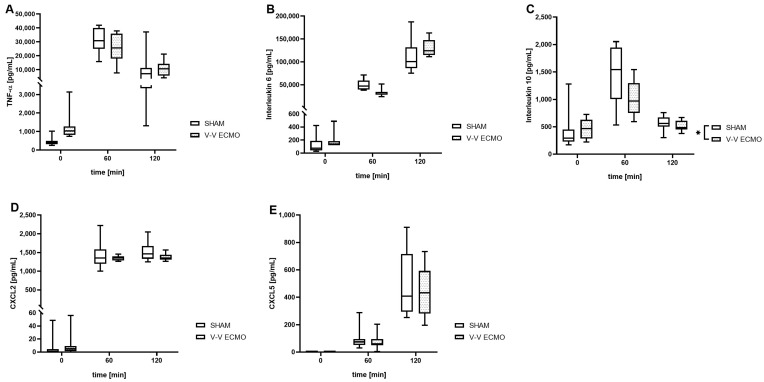
Time course of the concentration of the inflammatory parameters (**A**) tumor necrosis factor alpha (TNF-α), (**B**) interleukin 6 (IL-6), (**C**) IL-10, (**D**) C-X-C motif ligand 2 (CXCL2), and (**E**) CXCL5 in the plasma. While animals treated with V-V ECMO showed reduced concentrations of IL-10 compared to sham animals, no differences were measured regarding TNF-α, IL-6, CXCL2, and CXCL5. The asterisks denote the degree of statistical significance (* *p* < 0.05). Box and whisker plots indicate medians, interquartile ranges (boxes), and minimums and maximums (whiskers). Abbreviations: CXCL2 = C-X-C motif ligand 2; CXCL5 = C-X-C motif ligand 5; ECMO = extracorporeal membrane oxygenation; TNF-α = tumor necrosis factor alpha; V-V = venovenous.

Animals treated with V-V ECMO showed lower concentrations of interleukin 10 (IL-10; *p* = 0.046) compared to the sham group (Figure 4). However, no differences between the two groups were measured regarding tumor necrosis factor alpha (TNF-α; *p* = 0.831), IL-6 (*p* = 0.960), C-X-C motif ligand 2 (CXCL2; *p* = 0.237), and CXCL5 (*p* = 0.639; Figure 4).

**Figure 5:**
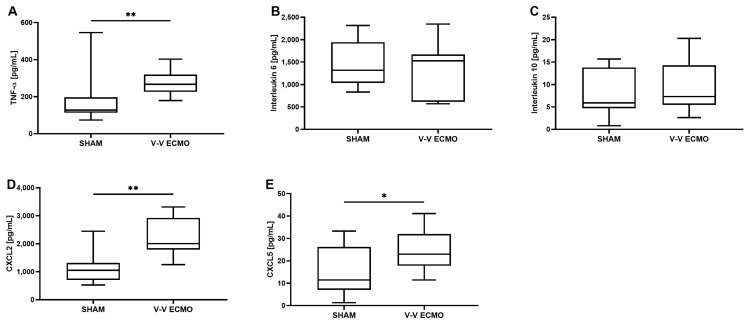
Concentration of the inflammatory parameters (**A**) tumor necrosis factor alpha (TNF-α), (**B**) interleukin 6 (IL-6), (**C**) IL-10, (**D**) C-X-C motif ligand 2 (CXCL2) and (**E**) CXCL5 in the bronchoalveolar lavage (BAL). Animals treated with V-V ECMO showed increased concentrations of TNF-α, CXCL2, and CXCL5. The asterisks denote the degree of statistical significance (* *p* < 0.05; ** *p* < 0.01). Box and whisker plots indicate medians, interquartile ranges (boxes), and minimums and maximums (whiskers). Abbreviations: CXCL2 = C-X-C motif ligand 2; CXCL5 = C-X-C motif ligand 5; ECMO = extracorporeal membrane oxygenation; TNF-α = tumor necrosis factor alpha; V-V = venovenous.

Furthermore, analysis of the bronchoalveolar lavage (BAL) revealed significantly elevated concentrations of TNF-α (*p* = 0.008), CXCL2 (*p* = 0.001), and CXCL5 (*p* = 0.035) after 2 h of V-V ECMO therapy compared to the sham group (Figure 5). However, no differences were seen between the two groups regarding IL-6 (*p* = 0.579) and IL-10 (*p* = 0.529; Figure 5).

## 3. Discussion

To the best of our knowledge, this is the first description of a live model of intestinal microcirculation during V-V ECMO therapy for septic shock in the rat with femoral drainage and jugular return. In brief, this study revealed three main findings. First, it demonstrated reduced SO_2_ and relative hemoglobin in the intestine and liver during septic shock and V-V ECMO therapy. Second, animals in the V-V ECMO group presented elevated systolic, mean, and diastolic blood pressures, with an increased heart rate, SV, CO, LVEDV, and LVEDP. Third, while decreased serum concentrations of IL-10 were measured during V-V ECMO therapy, increased concentrations of TNF-α, CXCL2, and CXCL5 in the BAL were captured in these animals.

Since several patients develop abdominal complications during V-V ECMO therapy and the measurement of the intestinal microcirculation is complicated in critically ill patients, the topic of this study is of great interest [12]. Our working group established the measurement of the hepatic and intestinal microcirculation by micro-lightguide spectrophotometry in a rat model of septic shock without V-V ECMO therapy [13]. Compared to healthy sham animals, reduced intestinal SO_2_ and blood flow were measured during septic shock. Furthermore, while hepatic SO_2_ and blood flow did not differ, a reduced relative hemoglobin level was observed during septic shock [13]. In contrast to this study, the animals were monitored for 3 h, and the microcirculatory measurements were analyzed as a percentage of the baseline. Given the lack of comparable studies during septic shock and V-V ECMO therapy, the results are compared with animal models of septic shock and humans during cardiopulmonary bypass. Albuszies et al. observed a preserved intestinal and increased hepatic oxygen saturation assessed per laser Doppler flowmetry in a model of septic shock in mice. Since the mice received continuous infusions of hydroxyethyl starch and norepinephrine, and septic shock was induced by cecal ligation, these results cannot be directly compared with our study [14]. Nahum et al. induced septic shock in piglets via lipopolysaccharide (LPS) infusion and measured reduced oxygen saturation in the liver with near-infrared spectroscopy, which is in line with our findings [15]. Unfortunately, no studies on hepatic microcirculation during V-V ECMO therapy are available. Therefore, the effect of the draining cannula on the hepatic oxygen saturation remains unclear. From a pathophysiological point of view, increased pressure on the inferior cava vein and hepatic veins should be avoided due to the multi-orifice design of the draining cannula, not affecting venous outflow.

Thorén et al. measured the jejunal mucosal perfusion using laser Doppler flowmetry in humans undergoing cardiopulmonary bypass (CPB). Interestingly, increased jejunal mucosal perfusion was measured 1 h after CPB [16]. It is worth considering that the heart-lung machine used during CPB cannot be directly compared with V-V ECMO. Besides the surgical impact on systemic inflammation, the effects of the suction pumps and the difference in the cannulation strategy cannot be ignored.

Bacterial translocation from the gut is caused by increased epithelial permeability [10]. Therefore, the microcirculation of the mucosa should be assessed. It cannot be neglected that the probe in this study was placed on the outside of the intestine rather than on the interesting mucosa inside the intestine. However, it must be underlined that the probes of the micro-lightguide spectrophotometry device are designed to measure oxygenation at a depth of between 2 and 4 mm [13]. Therefore, the results can be interpreted as a surrogate for intestinal microcirculation.

Microcirculation measurement is also realized by intravital microscopy [17]. However, the intestine has to be moved outside the abdominal cavity for reliable measurements. Although the vessels’ diameters can be easily measured, reliable quantification of tissue oxygenation is complicated with intravital microscopy. Further, microcirculation is affected by the surrounding conditions. Therefore, we decided to use micro-lightguide spectrophotometry to assess microcirculation because the intestine is moved back into its original position, minimizing its influence on perfusion.

Next, the hemodynamic data reflecting the macrocirculation were analyzed to find a potential reason for the reduced hepatic and intestinal microcirculation during V-V ECMO treatment in septic shock. Animals treated with V-V ECMO showed increased heart rates. The same results were seen by comparing sham and LPS animals without ECMO in our previous studies [13]. However, another study by Fujii et al., focusing on V-V ECMO in rats without septic shock, reported no differences between sham and ECMO animals regarding the heart rate [18]. Animals in the sham group in our study presented reduced systolic, mean, and diastolic blood pressures, which can be explained by respiratory acidosis in the sham group. In addition, LPS infusion is associated with systemic inflammation, vasodilation, and consequently, reduced systemic vascular resistance. These factors may also contribute to the reduced blood pressure in the sham group. Furthermore, animals treated with V-V ECMO showed increased SV, heart rate, and consequently, an increased CO. This, together with a reduced systemic vascular resistance, could explain the increased blood pressure compared to sham animals. However, increased levels of LVEDV were measured during V-V ECMO therapy, which could result from the increased cardiac rate. Nevertheless, the increased afterload could explain both the elevated LVEDV and LVEDP.

Interestingly, despite increased arterial blood pressure and CO, decreased hepatic and intestinal SO_2_ and relative hemoglobin were measured during V-V ECMO therapy in our study. Therefore, further analysis focused on blood gas analysis.

Animals in the sham group presented impaired pulmonary function reflected by reduced S_a_O_2_ and increased pCO_2_. A mixed respiratory and metabolic acidosis with decreased pH, base excess, and bicarbonate was also detected in animals in the sham group. As LPS is known to induce a cytokine storm, these changes could be caused by its infusion [19]. Interestingly, the positive effect of ECMO on S_a_O_2_ and pCO_2_ in the V-V ECMO group can be seen despite lung-protective ventilation with reduced frequency and tidal volumes, which underlines that the V-V ECMO model works well. As the ECMO circuit was primed with unbalanced hydroxyethyl starch containing 154 mmol/L of sodium and chloride, this fact may explain the measured electrolyte changes.

Further, dilutional anemia was also observed in the animals treated with V-V ECMO. It is worth noting that the critical hemoglobin concentration did not appear to be reached as no differences in the lactate concentration were measured between the two groups. Nevertheless, the dilutional anemia could have affected the SO_2_ measurements. Studies using cerebral near-infrared spectroscopy have shown that a reduced hemoglobin concentration is associated with reduced oxygenation measured by near-infrared spectroscopy [20]. Therefore, the impaired intestinal and hepatic SO_2_ measured in this study may be caused by the dilutional anemia of the ECMO circuit. Nevertheless, blood viscosity depends on the hematocrit, and therefore, the dilutional anemia could have improved the rheology of the blood. Riedel et al. investigated the oxygenation of the liver under different hematocrit values and found the best oxygenation at a hematocrit of 20% [21]. Since the measured hematocrit values during V-V ECMO therapy were even higher in our study, increased hepatic oxygenation and SO_2_ should have been expected compared to sham animals without hemodilution. Following this theory, Thorén et al. demonstrated that hemodilution during CPB in humans is associated with increased jejunal mucosal perfusion measured by endoluminal jejunal laser Doppler flowmetry [22]. In contrast to our study, humans without septic shock were analyzed during CPB. Therefore, these results cannot be transferred to V-V ECMO therapy during septic shock. To clarify this issue, new challenging animal studies must be designed with a sham group that receives dilutional anemia without changing the blood volume.

Due to the combination of ECMO-induced inflammation with septic shock, an increased inflammatory response could be expected during our experiments [6]. However, plasma analysis showed no differences between the two groups except for a reduced concentration of IL-10, which is known to suppress and terminate inflammatory immune responses largely through the inhibition of monocyte and macrophage activation, and is therefore also known to be a mediator of disease tolerance [23,24]. Tolerance reduces the negative impact of infection on host fitness and is also defined as a change in sensitivity to elicitors [25,26]. Tissue damage control involves the cell survival strategy of metabolic reprogramming, in which cells exposed to prolonged hypoxic conditions respond by decreasing oxygen demand [24,27]. According to the results of our study, disease tolerance seems to be suppressed. However, the kinetics of TNF-α, IL-10 and CXCL5 with a peak at 60 min reflect the design of our study with the administration of a single LPS bolus. A new study with a longer observation period and a continuous infusion of LPS is needed to clarify possible damage of the animals due to the suppressed disease tolerance. In this context, it must be noted that septic shock was induced by the injection of LPS from *Escherichia coli*. Therefore, these results cannot be directly transferred to a septic shock with bacteremia and the risk of infection of the membrane oxygenator [28,29].

Animals treated with V-V ECMO received lung-protective ventilation with a reduced tidal volume and respiratory rate. Therefore, reduced concentrations of inflammatory markers in the BAL could be expected. In contrast, increased concentrations of TNF-α, CXCL2, and CXCL5 were measured during V-V ECMO therapy in the BAL. Therefore, this study could show that V-V ECMO therapy during septic shock is associated with increased pulmonary inflammation measured via cytokine concentrations in the BAL. To interpret these results, further studies on rats treated with V-V ECMO without septic shock are needed. In the absence of prospective, matched, controlled studies, our results can only be compared with retrospective data analysis. Interestingly, a retrospective study by Zha et al. showed reduced 30-day mortality in humans with septic shock due to pulmonary infection treated with V-V ECMO compared to a matched control group [30]. However, no differences were seen regarding 90-day mortality. Thus, it could be hypothesized that V-V ECMO therapy is useful to treat acute hypoxia but the inflammatory damage to the lungs equalizes the long-term outcome in the context of septic shock. Consistent with this assumption, Falk et al. reported an in-hospital survival of 62.5% and a long-term survival of only 37.5% in septic patients with preserved left ventricular function treated with V-V ECMO [31]. Takauji et al. presented a retrospective multicenter study of septic patients treated with V-V ECMO and compared them with septic patients without ECMO therapy. No differences in 28-day or in-hospital mortality were observed. Interestingly, a sub-analysis of patients due to severe respiratory failure induced by lung infection showed a longer survival time compared to matched controls [32]. Thus, the source of sepsis seems to have an important impact on the mortality. However, it should be noted that all these studies reported on groups with less than 50 patients. Therefore, further multicenter studies are needed to clarify this issue.

This study has some limitations. First, results from animal studies cannot be directly transferred to humans. Nevertheless, rats have a very similar cardiopulmonary system to humans and offer the opportunity to form comparable groups with low variance due to inbred strains. Second, the administration of LPS from *Escherichia coli* results in a cytokine storm. Contrary to the sepsis-3 definition, no infection is present [4]. In this context, the puncture of the cecum with bacterial translocation to the abdominal cavity offers another model of septic shock. However, this model is challenging since animals need a second round of anesthesia and surgery. Further, the onset of septic shock is more heterogeneous than that with the infusion of LPS, and therefore, groups are more heterogeneous. Moreover, between the puncture of the cecum and the final experiments, the animals have to be monitored, which requires time, personnel, and money. It should also be noted that this procedure is often not authorized by the regional council for animal welfare reasons. For the same reason, the sample size in this study was kept small. Before more animals are included, this pilot study aimed to determine if the experimental design is reasonable. Based on the significant results, the inclusion of further animals in future studies is justifiable.

## 4. Materials and Methods

### 4.1. Animals

All procedures involving animals were conducted in accordance with animal care standards and the Animal Research: Reporting of In Vivo Experiments (ARRIVE) guidelines and approved by the local committee responsible for animal care (Animal Welfare Commission of the Department of Veterinary Medicine at the Regional Council of Giessen: GI 20/26 Nr. G 77/2019, Regierungspraesidium Giessen, Germany).

Male Lewis rats (330–350 g) obtained from Janvier Labs (Le Genest St. Isle, France) were kept at 22 °C, 55% relative humidity, and a day/night cycle of 14/10 h, with access to standard chow and water ad libitum. The animals were randomly divided into two groups per lot to receive V-V ECMO therapy or a sham procedure (*n* = 10 each). All cannulas were inserted into the vessels, and the rats were monitored for 2 h without V-V ECMO support during the sham procedure.

### 4.2. Induction and Maintenance of Anesthesia

As described previously, rats were orotracheally intubated (16 G cannula, B. Braun, Melsungen, Germany) and ventilated in a weight-adjusted and volume-controlled manner (tidal volume = 6.2 mL × body weight (kg)^1.01^, respiratory rate = 53.3 × body weight (kg)^−0.26^; Harvard Inspira, Harvard Apparatus, Cambridge, UK) with an inspiratory oxygen fraction (F_i_O_2_) of 0.5 after inhalative induction in an induction chamber (5% Isoflurane [Baxter, Unterschleißheim, Germany] balanced with 95% oxygen) [33,34]. Rats were placed on a heating pad, which was adjusted based on the temperature measured by a rectal probe. They were also provided heat with an infrared lamp to regulate the rectal temperature between 36.5 and 37 °C.

Next, the electrocardiogram was connected, and the lateral tail vein was cannulated percutaneously for the continuous infusion of fentanyl (10 µg/kg/h, Albrecht GmbH, Aulendorf, Germany), midazolam (2 mg/kg/h, Roche, Basel, Switzerland), pancuronium (0.1 mg/kg/h, Inresa, Freiburg, Germany), and a balanced crystalloid solution (5 mL/kg, Sterofundin B. Braun, Melsungen, Germany).

### 4.3. Cannulation and Abdominal Laparotomy

The following vascular accesses were placed surgically. First, a small median incision was made in the tail and the tail artery was dissected and cannulated with a 24 G cannula for the continuous measurement of the systolic, mean, and diastolic arterial blood pressure and intermittent blood gas analysis (B. Braun, Melsungen, Germany). Thereafter, a small incision was made in the neck, and the carotid artery and internal jugular vein were dissected. A 2 F pressure–volume catheter was carefully advanced through the carotid artery into the left ventricle to measure CO, SV, LVEDV, LVEDP, and LVEF (SPR-838, Millar, Houston, TX, USA). The internal jugular vein was cannulated with a modified, shortened ECMO return cannula connected to a three-way stopcock (20 G Surflo, Terumo, Eschborn, Germany).

After a median skin incision was made with scissors, the abdominal cavity was opened via electrocautery (AA01 Bovie high-temperature cautery, Bovie Medical Corporation, Clearwater, FL, USA). Next, the intestine was mobilized, and the vascular-free mesentery of a small bowel loop was dissected. The probe for the white light and laser Doppler spectrometry (LFX-151, LEA Medizintechnik GmbH, Heuchelheim, Germany) was then placed on the bowel and secured with a one-side-open silicone tube (diameter 8 mm) to assure a loose fit. The entire bowel was returned to its original position. Then, a shallow well was placed on the right lobe of the liver (LFX-45, LEA Medizintechnik GmbH, Heuchelheim, Germany). The abdominal cavity was covered with a warm, wet compress.

After a small skin incision in the inguinal region, the femoral vein was dissected and cannulated with a modified multi-orifice 64 mm cannula connected to a three-way stopcock (18 G Surflo, Terumo, Eschborn, Germany) for venous outflow to the ECMO circuit. Prior to cannulation, all animals received heparin through the lateral tail vein (400 IU/kg, Merckle GmbH, Blaubeuren, Germany).

### 4.4. Induction of Septic Shock

For the induction of septic shock, all animals received 1 mg/kg LPS of *Escherichia coli* O111:B4 (LPS-EB Ultrapure, InvivoGen, San Diego, CA, USA) via a syringe pump (11 Plus, Harvard Apparatus, Holliston, MA, USA) through the returning ECMO cannula over 30 min before commencing ECMO.

### 4.5. Extracorporeal Membrane Oxygenation

As previously described, the ECMO circuit consisted of a venous reservoir (M. Humbs, Valley, Germany), a roller pump (Verderflex Vantage 3000, Castleford, UK), and a membrane oxygenator (Micro-1, Kewei Rising Medical, Shenzhen, China) [33,34]. To avoid heat loss, a Heidelberger extension line was wrapped around the oxygenator and connected to a heating pump (B. Braun, Melsungen, Germany; HU35, Gettinge, Raststatt, Germany). The whole circuit was primed with 9 mL of hydroxy ethyl starch 6% (Voluven, Fresenius Kabi, Bad Homburg, Germany) and 250 IU of heparin (Ratiopharm, Ulm, Germany).

After carefully connecting the ECMO circuit to the three-way stopcocks of the drain and return cannulas, the blood flow was started at a rate of 45 mL/kg/min and then continuously increased to the target flow of 90 mL/kg/min. The sweep gas flow on the membrane was adjusted to between 20 and 70 mL/min to regulate the pCO_2_ between 35 and 45 mmHg. The oxygen fraction on the ECMO membrane was set to 0.5. For lung-protective ventilation, respiratory rate and tidal volume were set as 75% of the weight of the rat.

### 4.6. Intestinal Microcirculation

To measure intestinal microcirculation, the intestinal and hepatic probes were connected to the micro-lightguide spectrophotometry device Oxygen to See (LEA Medizintechnik GmbH, Heuchelheim, Germany). As previously described, each probe consisted of two different light sources and their corresponding optical sensors. White light spectroscopy (450–1000 nm) was used to measure the percentage of SO_2_, which is composed primarily of venous and secondarily of arterial and capillary oxygen saturation. The amount of light absorption induced by hemoglobin was analyzed and expressed in relative hemoglobin arbitrary units (RUs). The second light source emitted laser light (820 nm, 30 mW) determining erythrocyte velocity and consecutive relative blood flow [13].

### 4.7. Timepoints of Hemodynamic Measurements

Baseline values were captured before the administration of LPS. The subsequent measurements were recorded before the commencement of ECMO and every 10 min thereafter till 120 min.

### 4.8. Blood Analysis

Blood gas analyses were performed just before commencing ECMO and every 30 min thereafter till 120 min. The S_a_O_2_, S_cv_O_2_, pO_2_, pCO_2_, hemoglobin, hematocrit, pH, bicarbonate, base excess, lactate, glucose, sodium, potassium, calcium, and chloride were measured (ABL800, Radiometer, Copenhagen, Denmark). In addition, blood samples for inflammation analysis were taken just after starting ECMO and 60 min thereafter until 120 min. These samples were centrifuged at 5000 rounds per min for 5 min and the plasma samples were stored at −80 °C for further analysis.

### 4.9. End of Experiments

After 120 min, isoflurane was set to 5% and the animals were euthanized by exsanguination through the draining ECMO cannula. After confirming death by asystole, the neck was opened, and the trachea was dissected. Then, a ligature was fixed around the trachea to seal the tube. Next, the lungs were flushed repeatedly with a total of 40 mL of balanced crystalloid solution (Sterofundin, Fresenius, Bad Homburg, Germany). After the centrifugation of the BAL at 4 °C for 8 min at 1200 rounds per min, the supernatant was collected and stored at −80 °C for further analysis.

### 4.10. Enzyme-Linked Immunosorbent Assays

TNF-α, IL-6, IL-10, CXCL2, and CXCL5 were measured by enzyme-linked immune sorbent assays according to the manufacturer’s instructions (ELISA kits R6000B, RTA00, and R1000, R&D System, Wiesbaden, Germany; ELISA kits ERCXCL2 and ERCXCL5, Thermo Fisher Scientific, Waltham, MA, USA). Samples from the plasma and BAL were measured. The probes were thawed only once.

### 4.11. Statistics

All data are expressed as medians with 25th and 75th percentiles. Analysis of variance for repeated measurements followed by a post hoc Bonferroni test was used to analyze differences between the groups over time. Since the baseline values and first measurements were recorded without ECMO support, they were not included in the analysis of repeated measurements. Results of the BAL measurements were compared using the Wilcoxon–Mann–Whitney test. All statistical analyses were performed using SPSS Version 20 (IBM, Stuttgart, Germany). GraphPad Prism Version 7 was used for data presentation (GraphPad Software, San Diego, CA, USA). A *p* value < 0.05 was considered statistically significant.

## 5. Conclusions

In summary, rats treated with V-V ECMO presented impaired microcirculation of the intestine and liver during septic shock despite increased blood pressure and CO. Despite lung-protective ventilation, increased pulmonary inflammation assessed by BAL was observed during V-V ECMO therapy in septic shock.

## Figures and Tables

**Table 1:** Results of the blood gas analysis.

		0 Min	30 Min	60 Min	90 Min	120 Min
S_a_O_2_ *	sham	100 [99–100]	97 [96–98]	95 [94–96]	94 [92–95]	93 [92–95]
[%]	ECMO	100 [99–100]	100 [99–100]	98 [96–99]	96 [93–99]	94 [91–99]
S_cv_O_2_	sham	80 [77–85]	80 [74–86]	74 [69–79]	74 [70–75]	69 [64–71]
[%]	ECMO	72 [71–75]	78 [77–81]	74 [67–81]	73 [67–78]	63 [57–69]
pO_2_	sham	130 [115–140]	108 [106–120]	106 [96–110]	98 [90–100]	87 [79–94]
[mmHg]	ECMO	141 [130–148]	109 [103–126]	93 [83–107]	86 [71–104]	75 [62–87]
pCO_2_ ***	sham	44 [42–46]	58 [52–61]	60 [57–65]	61 [55–66]	60 [55–67]
[mmHg]	ECMO	44 [43–49]	39 [37–43]	40 [39–44]	43 [42–46]	37 [36–45]
pH ***	sham	7.37 [7.33–7.39]	7.25 [7.23–7.28]	7.21 [7.19–7.23]	7.23 [7.20–7.26]	7.25 [7.22–7.27]
	ECMO	7.36 [7.32–7.38]	7.41 [7.38–7.43]	7.38 [7.35–7.43]	7.38 [7.34–7.39]	7.44 [7.38–7.47]
Bic ***	sham	23.7 [22.8–25.0]	21.2 [20.5–22.1]	20.4 [19.2–21.0]	21.4 [20.3–22.7]	22.8 [21.9–23.3]
[mmol/L]	ECMO	24.2 [23.0–25.1]	24.8 [23.9–25.9]	24.1 [23.3–25.9]	24.7 [22.5–25.2]	25.6 [24.9–27.1]
BE ***	sham	−1.0 [−2.0–0.7]	−3.9 [−4.8–−2.7]	−4.8 [−6.5–−4.2]	−3.5 [−4.9–−2.0]	−1.8 [−2.9–−1.2]
	ECMO	−0.4 [−1.8–0.7]	0.5 [−0.7–1.7]	−0.5 [−1.3–1.6]	0.4 [−2.3–0.9]	1.5 [0.6–3.1]
Lac	sham	1.3 [1.1–1.5]	1.9 [1.8–2.1]	2.1 [1.8–2.8]	1.6 [1.4–2.2]	1.2 [1.0–1.7]
[mmol/L]	ECMO	1.4 [1.2–1.6]	1.9 [1.7–2.1]	2.6 [2.2–2.8]	1.9 [1.7–2.4]	1.3 [1.2–1.6]
Hb ***	sham	15.1 [15.0–15.5]	13.5 [13.2–13.9]	12.8 [12.3–13.3]	11.6 [11.2–11.9]	11.1 [10.3–11.5]
[mg/dL]	ECMO	15.2 [14.6–15.6]	8.8 [8.5–9.0]	8.4 [8.2–8.6]	7.6 [7.4–8.0]	7.3 [7.2–7.6]
Hct ***	sham	46.4 [46.0–47.4]	41.5 [40.6–42.6]	39.1 [37.9–41.0]	35.8 [34.6–36.6]	34.1 [31.8–35.4]
[%]	ECMO	46.4 [44.7–47.9]	27.3 [26.4–28.1]	26.0 [25.4–26.6]	23.7 [22.9–25.0]	22.6 [22.3–23.5]
Glu *	sham	163 [157–171]	213 [181–231]	184 [166–223]	147 [132–160]	138 [120–150]
[mg/dL]	ECMO	161 [154–171]	178 [174–185]	171 [161–176]	133 [115–143]	131 [128–135]
Na	sham	141 [141–142]	143 [142–143]	143 [143–144]	144 [142–145]	144 [143–144]
[mmol/L]	ECMO	142 [141–143]	143 [142–144]	144 [143–144]	144 [144–146]	144 [144–144]
K **	sham	4.5 [4.5–4.7]	3.9 [3.8–4.2]	3.9 [3.7–3.9]	4.2 [ 4.0–4.4]	4.6 [4.4–4.8]
[mmol/L]	ECMO	4.3 [4.3–4.4]	4.0 [3.9–4.0]	3.6 [3.5–3.6]	3.8 [3.7–3.9]	4.1 [4.0–4.2]
Cl *	sham	109 [108–110]	110 [109–111]	111 [110–112]	111 [111–113]	112 [111–114]
[mmol/L]	ECMO	109 [108–109]	111 [110–113]	113 [112–114]	113 [113–115]	114 [113–114]
Ca *	sham	1.51 [1.50–1.53]	1.47 [1.45–1.49]	1.46 [1.45–1.50]	1.46 [1.40–1.48]	1.43 [1.41–1.44]
[mmol/L]	ECMO	1.50 [1.48–1.56]	1.45 [1.44–1.47]	1.42 [1.37–1.45]	1.43 [1.38–1.46]	1.41 [1.38–1.42]

Data are presented as medians with 25th and 75th percentiles. The asterisks denote the degree of statistical significance (* *p* < 0.05; ** *p* < 0.01; *** *p* < 0.001). Abbreviations: BE = base excess; Bic = bicarbonate; Ca = calcium; Cl = chloride; ECMO = extracorporeal membrane oxygenation; Glu = glucose; Hb = hemoglobin; K = potassium; Lac = lactate; Na = sodium; pCO_2_ = arterial partial pressure of carbon dioxide; pO_2_ = arterial partial pressure of oxygen; S_a_O_2_ = arterial oxygen saturation; S_cv_O_2_ = central venous oxygen saturation.

## Data Availability

The original contributions presented in the study are included in the article, further inquiries can be directed to the corresponding author.
